# E3 Ubiquitin ligase RNF126 regulates the progression of tongue cancer

**DOI:** 10.1002/cam4.771

**Published:** 2016-05-26

**Authors:** Lina Wang, Xin Wang, Yuehan Zhao, Weidong Niu, Guowu Ma, Wei Yin, Chun Shi

**Affiliations:** ^1^ Department of EndodonticsCollege of StomatologyDalian Medical UniversityDalian116044China; ^2^ The State Key Laboratory Breeding Base of Basic Science of Stomatology (Hubei‐MOST) & Key Laboratory of Oral Biomedicine Ministry of EducationSchool & Hospital of StomatologyWuhan UniversityWuhan430072China

**Keywords:** Cell proliferation, E3 ubiquitin ligase, RNF126, tongue cancer, tumor burden

## Abstract

This study aims to analyze the role of RNF126 in the oncogenesis of tongue cancer. The cell proliferation and viability of human tongue cancer cells, SCC25 and SCC9 cells, were determined by cell counting and MTT assay, respectively. The effect of RNF126 on regulating AKT signaling pathway was analyzed through western blotting. The transplantation tumor model of nude mice was used to evaluate the tumorigenecity of RNF126. Knockdown of RNF126 inhibited the proliferation and viability of SCC9 and SCC25 cells. Inhibition of RNF126 also decreased the activity of AKT1 as well as its downstream molecules. Furthermore, RNF126 regulated the tumor volume on mice model. These data suggested that RNF126 might be related to the progression of tongue cancer through regulating AKT signaling pathway.

## Introduction

Posttranslation modification of protein which includes ubiqutination and phosphorylation is critical to activate the protein and maintain its function. Ubiqutination recruits a catalytic reaction to ubiquitinate the substrate 1. Three ubiquitin enzymes, E1 ubiquitin activating, E2 ubiquitin conjugating, and E3 ubiquitin ligase are sequentially recruited in this cascade reaction 2, 3, 4. Ubiquitin‐proteolytic enzyme system is one of the key regulators in protein degradation. The aberrant accumulation of oncoprotein which results from the abnormal proteolysis, contributes to the origination and progression of cancer. Previous studies have suggested that E3 ubiquitin ligase were involved in the occurrence of lung cancer, breast cancer, and oral cancer 5, 6.

Oral cancer ranks in the most relevant tumor death around the world. Although the 5‐year survival rate was gradually increased, great efforts are still needed in elaborating the mechanism underlying it, making early diagnosis, and applying the precision treatment. Precision medicine, which relied on the tumor biological features of patient focuses on the personalized treatment. It has already succeeded in treating cancer patients. Lung cancer patients with epidermal growth factor receptor (EGFR) mutation did respond well to the application of tyrosine kinase inhibitor (TKIs)^7^.

In order to find the oral cancer‐sensitive E3 ubiquitin ligases, we screened the human E3 ubiquitin ligase library and found that RING finger protein 126 (RNF126) might be the potential molecule involved in the occurrence of tongue cancer. RNF126 is an RING type E3 ligase which contains a Zn finger near the N‐terminus and a RING finger at the C‐terminus 8. It expresses in both the nucleus and the cytoplasm and is involved in a diverse set of cellular processes.

In this study, we further analyzed the role of RNF126 in the oncogenesis of tongue cancer. The growth and viability of human tongue cancer cells were inhibited with the knockdown of RNF126. The activity of AKT signaling pathway was also increased with the overexpression of RNF26. The knockdown of *RNF126* attenuated the tumor progression in vivo.

## Materials and Methods

### Cell culture and plasmid construction

Human tongue cancer SCC9 and SCC25 cells were maintained in DMEM medium with the supplement of 10% FBS. The RNF126 and RNF126‐RNAi was constructed according to the instructions of molecular cloning a laboratory manual. The efficiency of RNF126‐RNAi was determined by the immunoblotting analysis.

### The stable RNF126 knockdown SCC9 and SCC25 cells

The HEK293T cells were used as the retrovirus packaging cells. The packaging plasmids (pGAG‐Pol and pVSV‐G) together with RNF126‐RNAi retroviral plasmid were transfected into HEK293T cells for 24 h. Then the cells were incubated with new medium without antibiotics for another 24 h. The cell culture medium which was collected and filtered with 0.22 μm filter was added to SCC9 and SCC25 cells with polybrene (10 mg/mL). The retrovirus infected cells were treated with puromycin (0.5 mg/ml) for 7 days before the following experiments.

### Cell proliferation and viability

The stable RNF126 knockdown SCC9 and SCC25 cells (2 × 10^4^) were seeded on the 24‐well plates. The cell growth was determined through cell counting at day 2. For cell viability assay, the stable RNF126 knockdown SCC9 and SCC25 cells (2 × 10^3^) were seeded on the 96‐well plates. Then it was determined by 3‐ (4,5‐dimethylthiazol‐2‐yl)‐2,5‐diphenyltetrazolium bromide (MTT) at day 2, 4, and 6.

### Immunoblot analysis

Cells from 10‐cm cell dish were lysed in l mL Nonidet P‐40 lysis buffer (20 mmol/L Tris‐HCl, PH 7.4–7.5, 150 mmol/L NaCl, 1 mmol/L EDTA, 1% Nonidet P‐40, 10 *μ*g/mL aprotinin, 10 *μ*g/mL leupeptin, and 1 mmol/L phenylmethylsulfonyl fluoride). The detected molecules were fractionated by SDS/PAGE electrophoresis and subsequent immunoblot analysis was performed with the indicated antibody.

### Xenograft models

A total of sixteen 8‐week‐old male athymic immunodeficient Balb/c nude mice were purchased from Shanghai laboratory animal center. The stable RNF126 knockdown SCC9 or SCC25 (3 × 10^7^) cells were injected subcutaneously. Tumor diameters were recorded every 3 days. Specimens were harvested at 21 days.

## Results

### Knockdown of RNF126 inhibits the growth and viability of SCC9 and SCC25 cells

**Figure 1:**
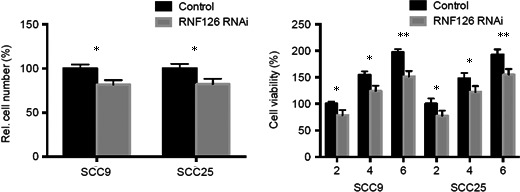
RNF126 regulates the proliferation and viability of SCC9 and SCC25 cells. Left: Knockdown of RNF126 inhibited the cell growth of SCC9 and SCC25 cells at day 2. Right: Knockdown of RNF126 inhibited the cell viability of SCC9 and SCC25 cells since day 2. *P < 0.05, **P < 0.01.

To confirm the role of RNF126 in tumor development, we first investigated the results of cell proliferation changes from the knockdown of RNF126. Both SCC9 and SCC25 cells showed significant decrease compared to the control group (Fig. 1). Then we monitored the cell viability changes of SCC9 and SCC25 cells. Knockdown of RNF126 consistently inhibited the cell viability since day 2 (Fig. 1). There was no significant difference between SCC9 and SCC25 cells. Based on the above results, we concluded that RNF126 regulated the cell growth and viability of human tongue cancer cells, SCC9 and SCC25 cells.

### RNF126 regulates the AKT signaling pathway

**Figure 2:**
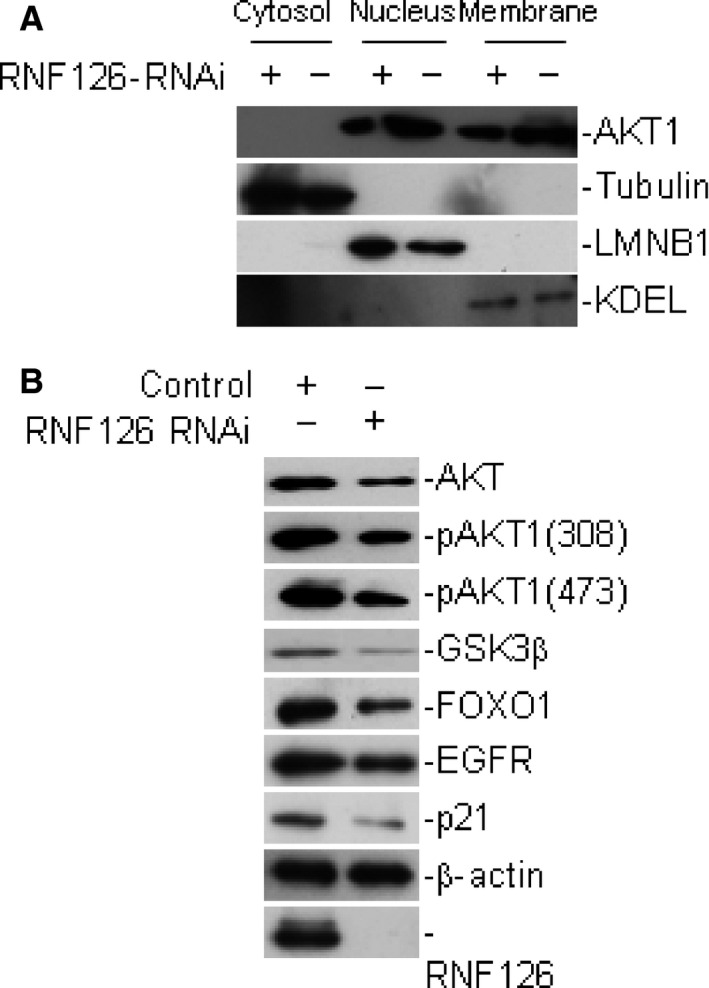
RNF126 participated in the AKT signaling pathway. (A) The transition of AKT1 into the nucleus decreased when RNF126 was knockeddown. (B) RNF126 regulated the phosphorylation of AKT1.

Previous study suggested that RNF126 interacted with the EGFR through a ubiquitin‐binding zinc finger domain and promoted the ubiquitylation of EGFR 9. To determine the function of RNF126 in tongue cancer development, we focused on the downstream signaling pathway of EGFR. We detected the expression changes of PI3K‐AKT signaling pathway, the STAT3 signaling pathway, the MAPK and ERK signaling pathway. The results suggested that PI3K‐AKT signaling pathway had significant changes when RNF126 was inhibited. The transition of AKT1 into the nucleus and the phosphorylation of AKT1 were decreased when RNF126 was knocked down. We also observed the attenuated phosphorylation of GSK3 and FOXO1 (Fig. 2). Therefore, we confirmed that RNF126 was involved in the AKT signaling pathway.

### RNF126 inhibits the tumor burden in vivo

**Figure 3:**
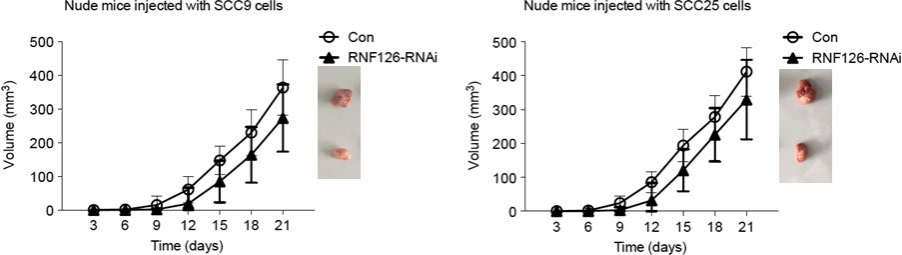
Knockdown of RNF126 alleviated the tumor burden of transplantation tumor model of nude mice which was originated from the SCC9 cells (left) and SCC25 cells (right).

The transplantation tumor model of nude mice was used to evaluate the tumorigenicity of RNF126. During the observation periods, the volume of subcutaneous tumor which originated from the stable RNF126‐knockdown tongue cancer cells was smaller than that from the control group (Fig. 3).

## Discussion

Tumorigenesis of tongue cancer, which is a complicated biological process, involves the active participation of genetic and environmental factors. It ranks on the most relevant health hazards because the current early detection rate is still pretty low. With the health education on tongue cancer prevention, the prevalence of environmental factors‐induced tongue cancer decreased. The focus of treatment and research has transferred to genetics and proteomics. The tumorigenesis ability of E3 ubiquitin ligase is also emerging with defined biological activities. Several E3 ubiquitin ligase, including NEDD4 5, 10, ITCH 11, WWP2 12, 13, and FBXO32 6 have been confirmed to be related with the tumorigenesis. In order to find the tongue cancer‐related E3 ligases, we screened the human E3 ubiquitin ligase library and found the candidate molecule, RNF126.

There are already several evidences that RNF126 is related to the tumorigenesis. First, the substrates of RNF126, like p21 14, Bag6 15, and EGFR 9 are involved in oncogenesis. It promotes cell proliferation partially through degrading p21 14. Second, it shares similar protein domains and structure with the E3 ligases, BCA2, which is involved in the progression of breast cancer 16. RNF126 was speculated to have similar function with BCA2. Furthermore, RNF126 was also confirmed that it highly expressed in a subset of breast cancer cell lines 17.

In this study, we contributed to determine the role of RNF126 in the development of tongue cancer. Firstly, we confirmed that RNF126 affected the proliferation of tongue cancer cells. Combined with the previous reported similar results, we concluded that RNF126 nonspecifically regulated the growth of cancer cells. Then, we observed that the knockdown of RNF126 also inhibited the cell viability of tongue cancer cells.

To elucidate the mechanism underlying these observations, we focused on the substrate of RNF126. RNF126 regulated the ubiquitin‐dependent sorting of cell surface receptors, EGFR, through the endocytic system. We studied the effect of RNF126 on the downstream molecules of EGFR. The changes in AKT signaling pathway were obvious. The transition of AKT1 into the nucleus and the phosphorylation of AKT1, the hallmarks of AKT1 activation, were inhibited with the knockdown of RNF126. Furthermore, we confirmed the tumorigenicity of RNF126 in nude mice. We speculated that RNF126 promoted the tumorigenesis through activating AKT signaling pathway. As the expression of PI3K did not show significant changes when RNF126 was knocked down, we thought that RNF126 might have substrate other than EGFR in this signaling pathway.

In conclusion, our data clearly established the role of RNF126 in the oncogenesis of tongue cancer. It provided a new strategy for developing treatment plan for tongue cancer. Further study is needed to determine whether RNF126 is a potential treatment target for tongue cancer.

## Conflict of Interest

None declared.
